# Transactions of the American Medical Association for 1854

**Published:** 1854-12

**Authors:** 


*Transactions of the American Medical Association for* 1854.—We have received the "Transactions" for the current year. As yet we have only had time to glance at its table of contents, and to place it side by side with the last year's volume for purposes of comparison.

The new publishing committee have certainly improved very much upon the preceding volumes. This one is bound in a neat and durable style, and makes altogether quite an ornamental appearance. The typography is excellent, the paper somewhat lighter than last year, but firm, clean, and smooth, and the impression is considerably better than heretofore.

The whole makes a volume of some 670 pp. In the table of contents we find the Minutes of the Seventh Annual Meeting; the Address of Dr. Parsons; the Report of the Committee on Medical Education; Report of the Committee on the Epidemics of Kentucky and Tennessee; an Essay on Erysipelas; the Medicinal Properties of the Cryptogamia; a Report on the Epidemics of Ohio, Indiana, and Michigan; another on those of Louisiana, Mississippi, Arkansas, and Texas; the Prize Essay; Report on the Norwalk Disaster; Dr. Linton's Remarks on Yellow Fever; Report of the Committee of Publication; Report of the Treasurer; Catalogue of Officers and Members; and indices.

Many of these papers evince considerable labor and research, and our impression is that the literary merits of the volume will compare favorably with those of any of its predecessors. This *should* be the case. As the Association grows older and the workings of its machinery become systematized, we should have a steady improvement in the character of its productions.

We have a living faith in the efficacy of the Association for good. All the whinings and complaints we have heard against it have not convinced us that it is not now a worthy exponent of the great profession it represents. There is no reason why its Transactions should not assume a leading importance in Medical literature.

We shall have occasion, from time to time, to speak of the merits of the various reports and essays, and to give to each a careful examination. H.

